# A Fiber Bragg-Grating-Based Miniature Sensor for the Fast Detection of Soil Moisture Profiles in Highway Slopes and Subgrades

**DOI:** 10.3390/s18124431

**Published:** 2018-12-14

**Authors:** Dingfeng Cao, Hongyuan Fang, Fuming Wang, Honghu Zhu, Mengya Sun

**Affiliations:** 1 School of Civil Engineering, Sun Yat-Sen University, Guangzhou 510006, China; wangfm6@mail.sysu.edu.cn; 2 College of Water Conservancy & Environmental Engineering, Zhengzhou University, Zhengzhou 450001, China; 3 National Local Joint Engineering Laboratory of Major Infrastructure Testing and Rehabilitation Technology, Zhengzhou 450001, China; 4 Collaborative Innovation Center of Water Conservancy and Transportation Infrastructure Safety, Zhengzhou 450001, China; 5 School of Earth Sciences and Engineering, Nanjing University, Nanjing 210023, China; smy@smail.nju.edu.cn

**Keywords:** highway slope and subgrade, fiber Bragg grating (FBG), aluminum oxide tube packed sensor (ATPS), temperature sensing, soil moisture

## Abstract

A fiber Bragg grating (FBG)-based aluminum oxide tube packed sensor (ATPS) was developed for the fast detection of the soil moisture profile in highway slopes and subgrades. The novel ATPS consists of an aluminum oxide tube with a diameter of 5 mm, an optical fiber containing a quasi-distributed FBG sensors, a “U”-shaped resistance wire, and a flange. There are four 0.9-mm diameter holes in the ATPS. Laboratory experiments were carried out to calibrate the relationship between the thermal response of ATPS and the soil moisture content. Two laboratory rainfall validation model tests were performed to validate the ATPS for capturing the soil moisture profile in highway slopes and subgrades. During the validations, the accuracy of the ATPS was quantified, and water infiltration through grassy and grassless ground surfaces were investigated. The calibrations indicate that the ATPS can detect and record real-time changes in the highway slope and subgrade moisture after rainfall, and reveal the most dangerous zones that occur at the connection between different construction materials. The average measurement accuracy of soil moisture monitoring was 0.015 m^3^/m^3^. Please note that the connection is where cracks form easily and the soil hydraulic conductivity increases significantly. The test results also indicate that grassy cover (lawn) significantly prevents water infiltration during the first few minutes of rainfall (twelve minutes in this study), after which, however, the infiltration rate drops sharply. The influence of lawn on water infiltration depends on the soil structure, hydraulic conductivity, and rainfall time. In summary, due to its small size and fast detection, the ATPS is a portable probe that can be used for moisture monitoring in highway slopes and subgrades.

## 1. Introduction

At present, water-related failures of subgrades and highway slopes are common around the world [1,2,3]. The instability of highway slopes and damage to subgrades have become one of the most frequent disasters in hilly and pluvial regions, threatening human life and leading to enormous monetary loss [4,5]. During periodic draining–recharging cycles, soil structures significantly deform in response to soil moisture changes, which consequently causes traffic accidents [6]. The slope and subgrade moisture level primarily depends on the season, especially in areas that have an obvious monsoon climate, such as China [3]. Therefore, accurate detection of the soil moisture of highways and subgrades in the rainy season and understanding the spatiotemporal variability are both helpful factors in estimating the rate of soil erosion to avoid casualties and reduce economic loss. 

Many soil moisture monitoring techniques have been developed. Su et al. [7] divided the soil moisture detection methods into 13 categories with respect to their characteristics, i.e., thermo-gravimetric method, time domain reflectometry (TDR), frequency domain reflectometry (FDR), the capacitance and frequency domain (CFD) technique, ground penetrating radar (GPR), remote sensing, infrared detection, and actively distributed temperature sensing (A-DTS). Dobriyal et al. [8] demonstrated that some methods yield less accurate results. The commonly used TDR can obtain accurate and fast-changing measurements. However, it is only applicable for small and shallow areas [8]. Compared with traditional point-type methods, A-DTS enables distributed and large-scale ground moisture measurement in the field, but the fiber optical cable must be installed into soil beforehand. Therefore, it is inapplicable for existing highway slopes and subgrades [9,10]. In addition, the installed optical fiber cannot be recycled, which will significantly increase the total instrument cost. For these reasons, it is imperative to develop a more powerful technique to measure soil moisture content profiles of highway slopes and subgrades. Most recently, a piezoceramic active sensing approach was developed to monitor soil moisture [11]. 

In geological, geotechnical, and environmental domains, the fiber Bragg grating (FBG) element was applied as a miniature detector for measuring humidity [12,13], strain and temperature [14,15,16], small deformations [17,18], and water or gas leaks [16,19,20,21]. The gas leakage location was inferred using a negative pressure wave method via FBG-based strain sensors [19,20]. Zhu et al. [22] concluded that FBG sensing systems have become a suitable tool for the monitoring and early warning of geo-hazards due to their inherent advantages of corrosion and electromagnetic interference resistance. Some researchers tried to detect soil moisture using the impact of changes in strain on the Bragg grating. Some special materials were coated around the Bragg gratings. These materials were sensitive to water content, which swell under the humid conditions and shrink in dry soils [12,13,23]. Kong et al. [24] developed an FBG-based bridge scour system using the moisture-sensitive polymers [21] that could swell to several times of the original volume upon the absorption of water. However, the sensors monitoring soil moisture via strain change is hard to applied in in situ projects for the limitation of optical fibers [25,26,27]. To improve the robustness of the polymer-coated FBG soil moisture sensors, Leone et al. [27] designed an ad-hoc house consisting of two independent plates to hold a hydrophobic microporous membrane. The FBGs were installed in the ad-hoc house. The gaseous water molecules could freely pass through the membrane. Although the feasibility of soil moisture monitoring using the thermo-hygrometers have been demonstrated, it is still unable to capture soil profiles as a probe for the limitation of cumbersome volume (more than 180 cm^3^). In order to reduce the size of FBG-based sensors and improve their robustness, Cao et al. [28] developed a special carbon fiber heated sensor (CFHS) that has been successfully applied in laboratory experimental studies. Carbon fiber itself is a corrosive agent and is a good candidate to generate Joule heat for the active thermal probing [29]. The CFHS detects the surrounding soil moisture content using thermal responses under the action of an electric current. A carbon fiber rod is used as a conductor to produce heat, and the temperature is recorded using the FBG. A thin polymer coating is packed outside the CFHS to protect the inner components. The CFHS is only fit for monitoring soil moisture in laboratory model tests. The CFHS must be fixed before soil model construction, instead of being inserted into soil, due to its weak coating and feeble rigidity and strength. Consequently, to quickly detect the in situ soil moisture of highways and subgrades using a low cost, portable, and miniature destructive tool, some further improvements should be conducted. 

In this study, an FBG-based aluminum oxide tube packed sensor (ATPS) was developed to detect highway slope and subgrade moisture. Compared with polymer materials, aluminum oxide has a higher electrical insulation capacity, thermal conductivity, and rigidity. Laboratory experiments were implemented to obtain the elementary parameters of the new designed probe. A highway model test was conducted to validate ATPS as a probe for fast soil moisture detection and hazard identification. The model experimental validations reveal the fact that ATPS can detect and acquire real-time changes in the highway slope and subgrade moisture after rainfall, and reveal the most dangerous zones that occur at the connection positions between different construction materials.

## 2. FBG-Based Portable Soil Moisture Sensor: Principles and Design

**Figure 1:**
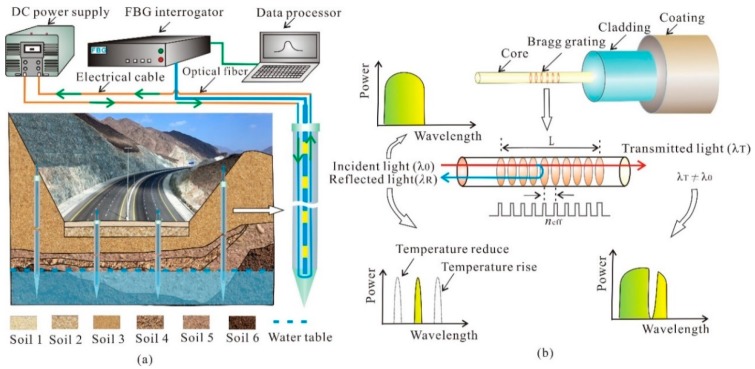
Schematics of the aluminum oxide tube packed sensor (ATPS) system: (**a**) basic configuration for fast detection of the subgrade and slope moisture content, and (**b**) temperature sensing principles of FBG. DC is direct current; *n*_eff_ is the valid refractivity (dimensionless); *L* is the total space of the gratings domains; and $\lambda_{0}$, $\lambda_{R}$, and $\lambda_{T}$ is the wavelength of pump incident ray, reflected ray, and transmitted ray, respectively.

The measurement principles result from the relationship between the thermal response of the temperature characteristic value (*T*_t_) and soil moisture. The *T*_t_ is defined as the average temperature during an effective period. During a measurement, an ATPS is inserted into the soil and then heated by a resistance wire in it. Under the action of a constant electric current, the temperature rises with elapsed time. Here, a portable FBG interrogator was employed to measure the soil temperature. According to the recorded temperature of the ATPS, the *T*_t_ is calculated from which the soil moisture (*θ*) is calculated [28]. Figure 1 presents the schematic details of the ATPS monitoring system. 

The central wavelength of the reflected ray meets Equation (1) [22,25]:

(1) $$\lambda_{B}=2n_{\mathrm{eff}}\Lambda$$

where $n_{\mathrm{eff}}$ is the valid refractivity (dimensionless), L is the total space of the gratings domains, and $\lambda_{B}$ is the central wavelength. The relationship between $\lambda_{B}$ drift and strain (or temperature) is given as [22]:

(2) $$\frac{\Delta \lambda }{\lambda_{B}}=(1-P_{\mathrm{eff}})\varepsilon_{z}+(\alpha_{T}+\zeta_{T})\Delta T$$

where ΔT is the temperature variation (°C), Δλ is the changes of $\lambda_{B}$ (nm), $P_{\mathrm{eff}}$ is the valid elastic index, $\varepsilon_{z}$ is the strain in the axial direction, $\alpha_{T}$ and $\zeta_{T}$ are the thermal expansion (1/°C) indexes [22].

When heating soil via the ATPS, the line power theory is used. The ATPS is presumed to be infinitely long and the diameter of the ATPS can be ignored. The temperature T meets the following relationship [30,31,32]:

(3) $$T=-\frac{q^{\prime }}{4\pi \lambda }\ln\left(\frac{4\kappa t}{r^{2}}-\gamma \right)+T_{0}$$

where $q^{\prime }$ is the heating power under a constant electrical current (J/ms), r denotes the gap between the temperature recording position and geometric center of sensors (m), t is the electric power time (s), *λ* represents the thermal conductivity of the soil surrounding the ATPS (W/m°C), k represents the thermal diffusivity (m^2^/s), γ denotes Euler’s constant, and $T_{0}$ represents the initial undisturbed temperature (°C). Sayde et al. [30] advised using a sectional function to calculate soil moisture based on temperature rise:

(4) $$\theta =\left\{ \begin{array}{ll} \exp\left(\frac{c_{1}+T_{t}}{c_{2}}\right)+c_{3} & \theta \geq \theta_{0}\\ kT_{t}+b & \theta <\theta_{0} \end{array}\right.$$

where $T_{t}$ is the average temperature during the valid period (min), and $\theta_{0}$ is the threshold value (m^3^/m^3^) [33]. $c_{1}$, $c_{2}$, $c_{3}$, k, and b are constants.

**Figure 2:**
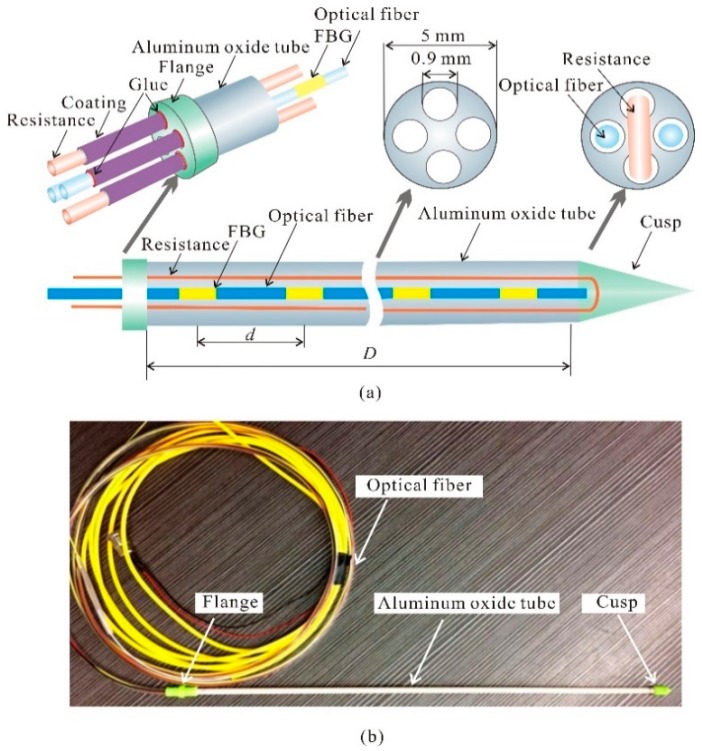
Details of the ATPS: (**a**) frame of the ATPS, and (**b**) images of the ATPS. *d* is the gap between each adjacent FBGs, and *D* represents the length of the ATPS.

To overcome the shortcomings of CFHS in in situ moisture monitoring, an improved sensor (ATPS) was designed, as shown in Figure 2. The fistulous ATPS is composed of an aluminum oxide tube, a series of FBG sensors imprinted on a multimode optical fiber, a “U”-shaped resistance wire, and a flange. There are four holes in the ATPS, and each one has a diameter of 0.9 mm, as shown in Figure 2. An electrical resistance wire with a dimeter of 0.85 mm is installed in two of the holes and presents a “U” shape. At the bottom of the “U”, a plastic cusp is fixed on the aluminum oxide tube using epoxy glue.

For the other two holes without resistant wires, an optical fiber with Bragg gratings is inserted into each hole. With the diameter limitation of the ATPS, traditional thermistors cannot be used to replace small-sized optical fiber here. To avoid the influence of strain, the bottom end of each optical fiber is free, and the space between each optical fiber and the flange is fixed using glue. The optical fibers and resistance wires exposed in air are packed using polymer coats to reinforce their strength and to prevent electrical leakage. During a measurement, the optical fibers should be collected with an FBG interrogator, and the resistance wires ought to be collected with a direct current power (DC). The electrical resistivity of the wire is 25.38 Ω/m. The separation distance between neighboring FBGs (*d*) can be set based on the application requirements; however, it cannot be less than 2 cm. The maximum number of FBGs on each optical fiber is 12, which is the limitation that the interrogator can distinguish at present. If more FBGs need to be installed, more optical-fiber-recorded FBGs should be inserted into the ATPS holes.

## 3. Laboratory Calibration

### 3.1. Experimental Setup

**Figure 3:**
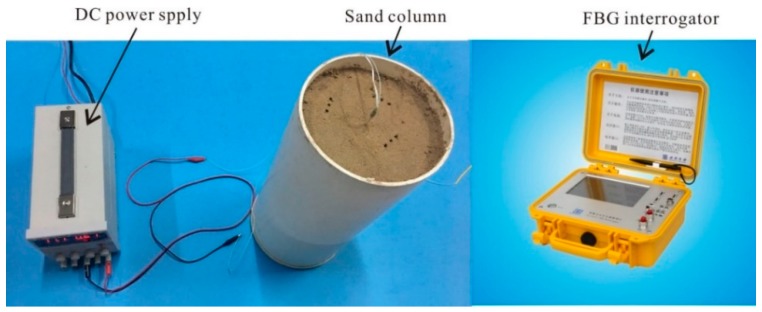
Photographs of the laboratory calibration devices.

To determine the relationship between θ and $T_{t}$, several laboratory calibrations have been finished. These experiments were conducted in a polyvinyl chloride (PVC) pipe (Figure 3). The length of the PVC column was 40 cm, and the diameter was 15 cm. One end of the PVC tube was closed and the other one was open.

**Figure 4:**
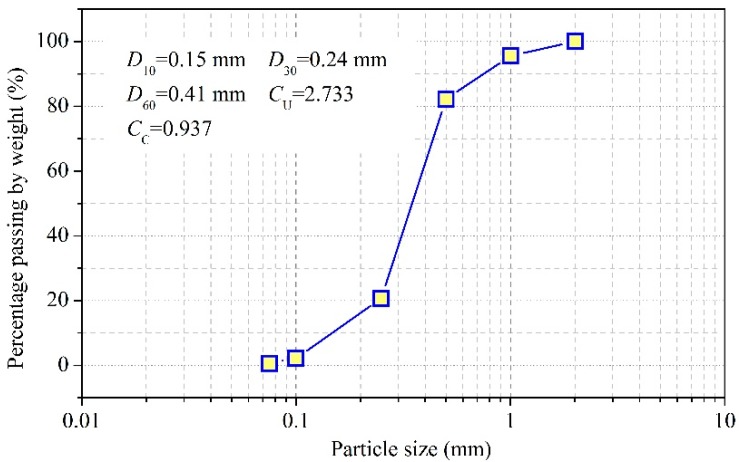
Grain size distribution of the soil used in the experiments. *D*_10_ (or *D*_30_ and *D*_60_) means that 10 (or 30 and 60) percent of the particles were finer and 90 (or 70 and 40) percentage of the particles were coarser than *D*_10_ (or *D*_30_ and *D*_60_). *C*_U_ is the uniformity coefficient that is the ratio of *D*_60_ over *D*_10_. *C*_C_ is the gradation coefficient, that is, the ratio calculated from (*D*_30_ square) over (*D*_60_ into *D*_10_).

Before the calibrations, the soil was oven dried under a high temperature condition (105 °C). The soil samples different volume of water poured into them. The prepared initial water contents were 0, 0.02, 0.04, 0.06, 0.08, 0.1, 0.14, 0.16, 0.2, 0.24, 0.28, 0.32, and 0.36 m^3^/m^3^. During the soil filling process, the ATPS was first fixed in the central position of the PVC column, and the test soil was then put into space around the ATPS. The ATPS used in the calibration test had a length of 40 cm and the distance between every two adjacent FBGs was 4 cm. There were 10 FBGs in total. The dry density of soil was 1.60 g/cm^3^. When a measurement began, the ATPS was connected to the DC power. The heating power was 2.21 W/m. The wavelength and values of temperature were inferred from the central wavelength demodulated by an interrogator (Model No. NZS-FBG-A03). The FBG wavelengths were recorded every 5 s. Figure 4 depicts the particle size distribution of the test soil. The saturated θ reached 0.352 m^3^/m^3^, and the saturated hydraulic conductivity was 3.13 × 10^−5^ m/s.

### 3.2. Results and Analysis of the Laboratory Calibrations

**Figure 5:**
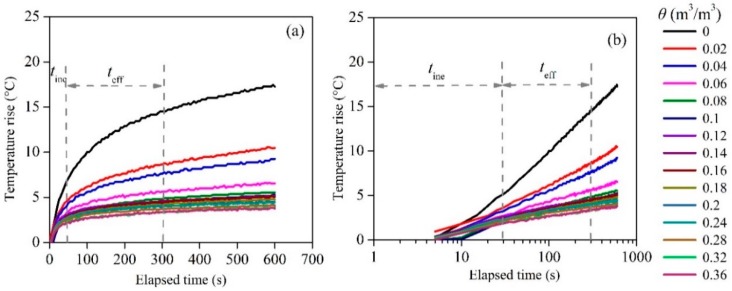
Temperature response versus the elapsed time: (**a**) average temperature response of the ATPS, and (**b**) relationship between the temperature rise and logarithmic time. $t_{\mathrm{eff}}$ is the effective time period, $t_{\mathrm{ine}}$ is the ineffective time period, and θ is the soil moisture content.

When establishing the relationship between θ and $T_{t}$, sufficient samples must be collected. In the in situ tests, Sourbeer and Loheide [34] used 12,673 points collected in two years when relating θ to $T_{t}$. However, during laboratory calibrations, increasing samples or measurement time was impractical because of high cost. Another way to enlarge the sample number in this study was by increasing the number of FBG sensors in the same calibration. Figure 5 shows the average temperature response versus the elapsed time. The average temperature was calculated from the temperatures of the 10 FBGs. Lhendup et al. [35] recommended discarding the temperature data from the initial seconds to avoid the presence of a transient temperature gradient and only use data that is unaffected by the sensor itself. The number of seconds to discard is dependent on the length of the period when the heat conduction gets to a steady state. According to the heat transfer characteristics, the heating period can be divided into the effective time period ($t_{\mathrm{eff}}$) and the ineffective time period ($t_{\mathrm{ine}}$). During $t_{\mathrm{eff}}$, the relationship between temperature rise and logarithmic elapsed time can be described linearly, as shown in Figure 5b. Here, $T_{t}$ was calculated form the 120th to the 300 th s. After each measurement, the ATPS was drawn out to cool down. The total cooling time was 180 s. The total soil moisture measurement time was 480 s that equals heating time plus cooling time.

**Figure 6:**
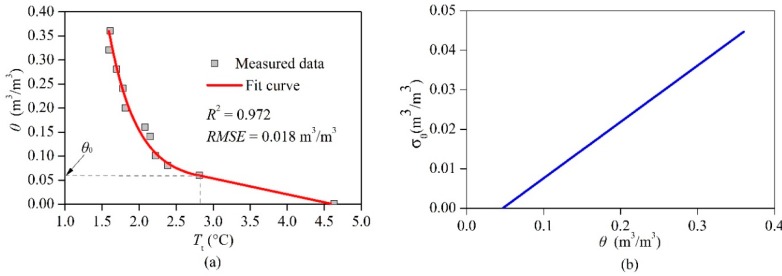
Laboratory calibration results: (**a**) fitted results between the soil moisture content and the temperature changes of the ATPS, and (**b**) the calculated error ($\sigma_{\theta }$) variation with the increase of the actually measured soil moisture content obtained by the oven drying method. *R*^2^ is the coefficient of determination of a piece functional regression, root mean square error (*RMSE*) is defined as the standard deviation of the residuals, and $T_{t}$ refers to the temperature characteristic value.

Figure 6a shows the calibrated relationships between θ and $T_{t}$. For the soil used in this study, the $\theta_{0}$ value was 0.06 m^3^/m^3^. The $\theta -T_{t}$ relationship can be expressed as:

(5) $$\theta =\left\{ \begin{array}{ll} \exp\left(\frac{1.1567-T_{t}}{0.3756}\right)+0.04675 & \theta \geq 0.06\\ -0.0329T_{t}+0.1525 & \theta <0.06 \end{array}\right.$$

The coefficient of determination (*R*^2^) was 0.972 and the root mean square error (*RMSE*) was 0.018 m^3^/m^3^ for the fitted results in Equation (5). It should be emphasized that when the soil was very humid, it was practically impossible to maintain a consistent soil water content; the soil column drained rapidly so that the bottom of the column was saturated while the top had a lesser soil water content value. Hence, both θ and $T_{t}$ were average values for the sand column. The diameter of the sand column depends on the heating power and time (for further details, refer to Reference [9]).

Sayde et al. [30] recommended using an error function ($\sigma_{\theta }$) to describe the measurement error of the heated cable. Cao et al. [28] successfully used $\sigma_{\theta }$ to estimate the errors of CFHS. It is defined as:

(6) $$\sigma_{\theta }=\frac{\sigma_{T_{t}}}{\left|\frac{df(\theta )}{d\theta }\right|},$$

where f(θ) is the function that was fit between $T_{t}$ and θ, and $\sigma_{T_{t}}$ (m^3^/m^3^) is the standard deviation of $T_{t}$ (°C). According to the calibrated Equation (5), the $\sigma_{\theta }$ for this study can be simplified and written as:

(7) $$\sigma_{\theta }=0.1426\theta -0.0067$$

The experimental data acquired by Sayde et al. [30] indicated that when the θ value was about 0.05 m^3^/m^3^, the $\sigma_{\theta }$ was 0.001 m^3^/m^3^, while when the θ was about 0.41 m^3^/m^3^, the $\sigma_{\theta }$ was 0.046 m^3^/m^3^. These conclusions agree well with the estimated error results of this study, as shown in Figure 6b. After investigating the impacts of the heating methods on the water content monitoring using the A-DTS method, Dong et al. [36] found that the susceptibility of A-DTS to the water content depended on the heating strategy; increasing the power input and heating time could improve the $\sigma_{\theta }$. It was also discovered that the ambient temperature seriously affected the $\sigma_{\theta }$ [36].

The effects of the ambient temperature on the estimations of ATPS can be described using a third-order polynomial equation [36]. Gamage et al. [37] revealed the relationship between the estimated error of the A-DTS method and the depth, and recommended calibrating the relationship between the temperature rise and the θ in the field. However, Sourbeer and Loheide [34] proposed that the estimated error of A-DTS was also influenced by previous soil wetting and drying cycles. To further investigate the stability, reliability, and accuracy of the ATPS, the calibrated results were verified through a validation test.

## 4. Laboratory Validation

### 4.1. Experimental Setup for the Validation Test

**Figure 7:**
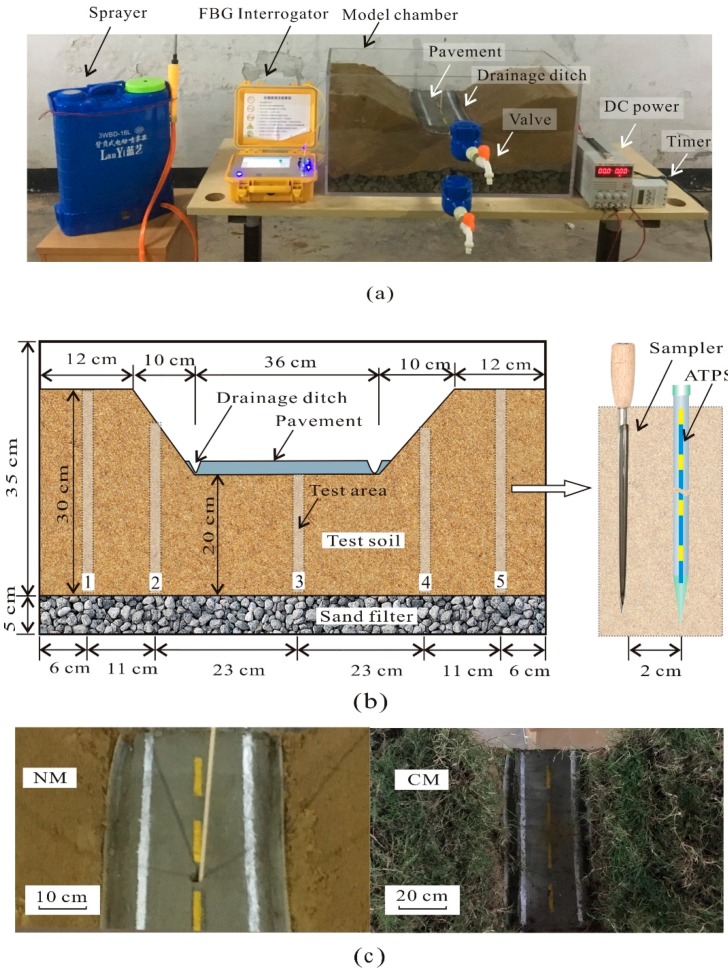
Setup of the validation experiments and instrumentation details: (**a**) photograph of the test setup, (**b**) front view of the model chamber, and (**c**) photos of NM and CM tests.

To verify the feasibility of ATPS, two laboratory validation tests were performed in an 80 cm × 40 cm × 40 cm cuboidal model chamber (Figure 7). The disturbed radius was about 5 cm, so the distance between adjacent ATPSs should be more than 10 cm to avoid mutual interference. In addition, the distance between boundaries and ATPSs should be more than 5 cm to avoid the influence of the chamber walls. Thus, the lowest horizon distance from central pavement to chamber walls was 20 cm (22 cm in this study, as shown in Figure 7). It is also important to point out that this validation test was only performed to justify the feasibility of highway slope and subgrade moisture profiles detection, rather than simulate an actual slope.

The device used to perform the validation test included a sprayer, an FBG interrogator, a model chamber, a DC power source, and a timer. The sprayer was used to simulate rainfall, and the rain intensity could be adjusted. The model chamber walls were made up of transparent acrylic resin with a thickness of 1 cm. In the model chamber, a highway model was constructed. Two soils were used to build the model; the upper test soil was the same as that adopted in the calibration tests, and the lower soil was coarse sand which acted as a filter to prevent movement of the upper soil during the experimental process. A DC power source was used to provide a constant electrical current. To automatically heat and collect data, a timer was applied, which controlled all of the measurements according to input orders. In the first test, the model soil was naked, and the rainfall directly infiltrated the soil (hereafter called the NM test), but in the second test, there was a grass cover on the model to prevent vertical infiltration (hereafter called the CM test).

Figure 7b shows the details of the highway model that included two slopes and a subgrade. On the surface of the subgrade, a concrete pavement was laid. Where the slope and pavement crossed, a drainage ditch was constructed. The ditch was a groove that was half of a polythene (PE) tube with an inner diameter of 15 mm. Valves were installed at the pavement and the bottom of the chamber. The drainage ditches were collected with the upper valve. All the parameters of the soil and ATPS in the validation tests were the same as those used in the calibration experiments.

The NM and CM tests included two stages. In the first (raining) stage, the spray was opened to produce a constant intensity rainfall. Here, the raining intensity was 0.4167 mm/min, and the total raining time was 40 min. During the raining process, the drainage water was collected using a graduated cylinder, and the volume was read every six minutes. After the rainfall, the test procedure entered into the second (no raining) stage, and soil moisture profiles were detected using the ATPS at different positions for different drainage times. The water infiltration and drainage in soil were presumed to occur from the beginning of the rainfall. Five test areas were chosen for the collection of soil moisture, as shown in Figure 7b. When determining the soil moisture profiles, the ATPS was inserted into the soil and then heated. After each measurement, the ATPS was drawn out and the borehole was backfilled with test soil with θ = 0.14 m^3^/m^3^. It should be pointed out that the later measurement positions were not located on the former backfilled locations to avoid the influence of backfilled materials.

After draining for 18 h during the NM test, soil columns were collected using a sampler from areas 1, 2, 4, and 5. The sampler cut the soil columns from the surrounding soil by artificial rotation to measure their θ using the oven drying method. Because it was easy to destroy the concrete pavement using the sampler, no soil column was collected from area 3. After the soil column collection, all of the soil in the model chamber was cleared away, and new soil was filled for the CM test. The model soil constructed for the highway slope and subgrade model was the same as that used in the NM test, as was the density and other physical parameters. On the surface of the model, a grass cover (lawn) was transplanted. To promote the growth of the grass, the rainfall test began 14 days later, and the following experimental process was the same as that of the NM test.

### 4.2. Validation Results and Discussion

**Figure 8:**
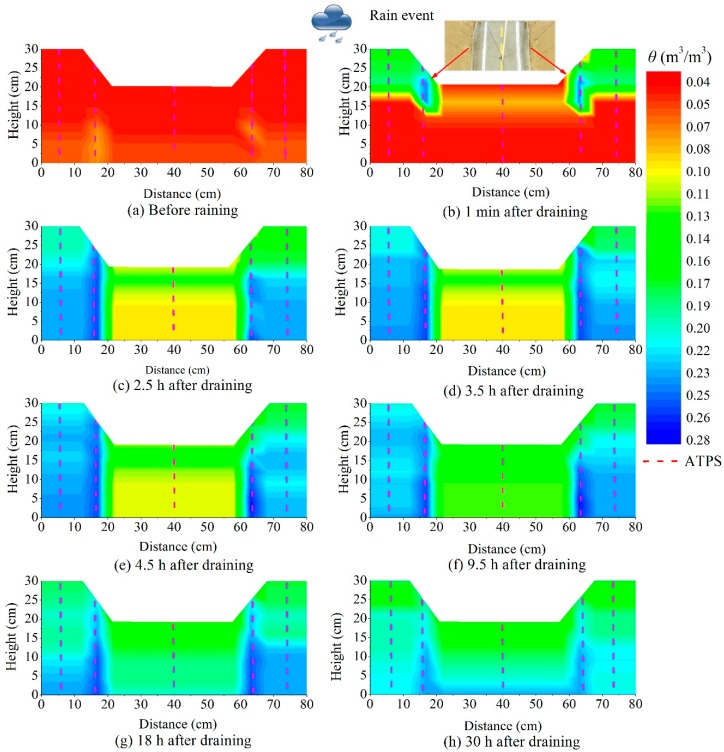
Soil moisture content (color) on the subgrade and slope after rainfall during the NM test.

Figure 8 depicts the soil moisture distribution at 1 min, 2.5, 3.5, 4.5, 9.5, 18, and 30 h after draining during the NM test. As shown in Figure 8b, the precipitation initially infiltrated into the connection position between the drainage ditch and the slope, which was because the PE tube and soil were different materials. After absorbing water, the soil strength and stress changed significantly, which led to obvious deformation, and water had less influence on the PE tube but could cause some cracks between these two materials [38]. Chen et al. [4] proposed that cracks increased the air space ratio, and the soil permeability. The flowing water in fractures weakens the foundation of the pavement, so assessing the moisture susceptibility among different mixtures is very important. Figure 8b shows that for the model test in this study, the most dangerous zone was the connection position, but for actual applications, this conclusion may be not true because there are often some revetments on the slope surface that prevent water infiltration. However, the phenomenon shown in Figure 8b indicates that the connection zones should be a focus of future studies.

**Figure 9:**
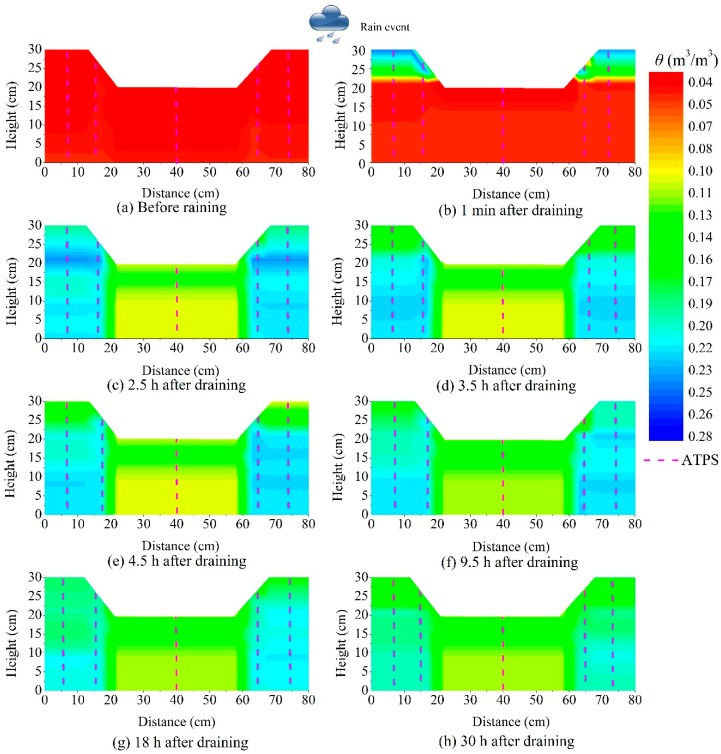
Soil moisture content (color) changes of the subgrade and slope after rainfall during the CM test.

Figure 9 shows the soil moisture distributions at 1 min, 2.5, 3.5, 4.5, 9.5, 18, and 30 h after draining during the CM test. In contrast to the soil moisture profiles from the NM test, there was no water leakage that occurred in the slope due to the grass lawn cover. At each moment during the draining period, the soil moisture in the NM test was larger than that in the CM test, which agrees well with the results obtained in Reference [39]. Chang et al. [40] used the soil moisture difference to optimize the prediction model. It should be stated that the soil moisture contours in Figure 8 and Figure 9 do not reflect the actual soil moisture distribution, especially under the pavement. In order to reduce the disturbance to the pavement, only one test area (area 3) was chosen to show how to gather data under the pavement and in actual applications. Nevertheless, the distance between each adjacent monitoring position depended on the geological and hydrological conditions.

**Figure 10:**
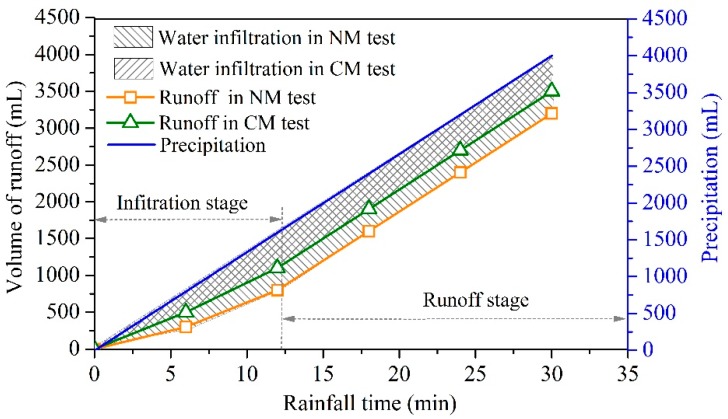
Relationship between the runoff volume, cumulative precipitation, and rainfall time.

The vertical infiltration model after rainfall could be divided into two types: unsaturated infiltration, in which the rainfall supply intensity does not reach the drainage ability of the soil; and ponding infiltration, when the rainfall rate is larger than the soil drainage rate [41]. The water transportation models for these two types are definitely different. For the NM and CM tests in this study, all of the infiltration happened under ponding conditions, and it was possible to describe the infiltration process by the Green–Ampt model [42]. The cumulative precipitation equals the sum of the infiltrated water plus the surface runoff. Therefore, the infiltration rate can be indirectly inferred from the runoff. Figure 10 shows the measured runoff and the cumulative precipitation measured by the graduated cylinder.

The cumulative precipitation can be calculated using:

(8) $$V=ISt_{1}$$

where V is the cumulative precipitation (mL), I is the rain intensity (mm/min), S is the cross sectional area that was calculated by multiplying the length by the width of the model chamber (cm^2^), and $t_{1}$ is the draining time. Here, after substituting I = 0.4167 mm/min, S = 3200 cm^2^, and $t_{1}$ = 6 min into Equation (8), V = 133.33 mL/min was obtained. As shown in Figure 10, the relationship between the runoff volume and the rainfall time can be divided into two stages according to the slopes: the infiltration and runoff stages. During the first twelve minutes (infiltration stage), the volume of runoff in the NM test was less than that of the CM test, which means more water infiltrated the soil during the NM test. After twelve minutes of rainfall, the slope of the volume of runoff in the NM and CM models did not change with time, and therefore a cumulative precipitation slope of V = 133.33 mL/min was maintained. Thus, it can be concluded that the infiltration during the runoff stage was negligible. Mao et al. [43] proposed that the infiltration rate decreased sharply in the first 12 min of infiltration in accordance with the original and modified Green–Ampt models.

**Figure 11:**
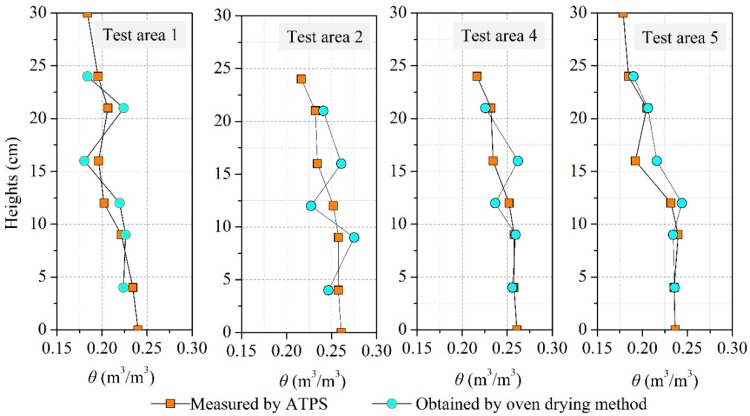
Soil moisture profiles measured 18 h after rainfall by two methods.

For the sake of evaluating the reliability of the data acquired by the ATPS, the soil profiles measured using the ATPS and the oven drying method were compared (Figure 11). It can be observed from Figure 11 that in test areas 1, 4, and 5, the soil profiles measured by the two methods agree well with each other, which reflect the same subsurface moisture distribution trends. In test area 2, the soil moisture profile detected by the ATPS was smoother than that of the oven drying method, which was primarily due to the loss of water during oven drying.

**Figure 12:**
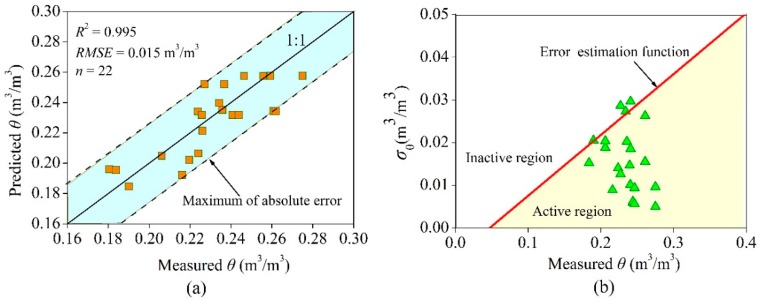
Error analysis of the ATPS: (**a**) absolute error analysis, and (**b**) error estimation function assess.

Figure 12 shows the errors of the ATPS. Regarding the data acquired using the oven drying method as the true values, the error of the ATPS was evaluated. In Figure 12a, most of the points are located near the 1:1 line, with an *RMSE* of 0.015 m^3^/m^3^ and an *R*^2^ of 0.995, which was suggested as effective [44,45,46]. Figure 12b shows the errors calculated using $\sigma_{\theta }$. The error estimation function line divides the entire region into the upper inactive region and the lower active region. If a measured datum is located in the active region, that means the defined error estimation function was effective, and it enables the description of the error change with soil moisture. Twenty-two samples were collected, and only 20 points were in the active region (90.1% were effective), which demonstrates that the $\sigma_{\theta }$ function was effective [44,45,46].

Although the feasibility of highway slope and subgrade moisture detection using the ATPS had been demonstrated in laboratory tests, there were still two obstacles to overcome. The first one was that the maximum depth that the ATPS could reach was still unknown. In this study, the maximum depth used was only 40 cm; however, this depth cannot meet the actual application requirements. In theory, the maximum depth depends on many factors, such as the diameter of the ATPS, the soil density, and the maximum number of FBGs. Hence, more field experiments are necessary to further validate the performance of the ATPS. The second obstacle came from the laboratory calibrated relationship between θ and $T_{t}$.

When analyzing in-situ water content profiles in the field, the key soil layers must be considered, such as the weak intercalated layer, gravel layer, and impermeable layer. In the homogeneous model, the soil moisture content under the pavement changed slightly without the influence of the key soil layers. However, in field sites, these key soil layers can change water infiltration paths. Hence, if some cracks were detected using the ATPS, besides the soil moisture profiles, additional considerations are necessary to analyze the safety of highways. In addition, the rainfall time should be investigated for field applications, because it directly affects the water infiltration model.

## 5. Conclusions

An FBG-based method for monitoring highway slope and subgrade moisture was introduced and the aluminum oxide tube packed sensor (ATPS) was designed in this paper. The details of the ATPS was presented. The parameters of the ATPS were obtained in a laboratory experiment. Two laboratory rainfall validation model experiments were performed to validate the ATPS. The conclusions are summarized as follows:

(1) The ATPS indirectly infers soil moisture via the heat transfer rate from the ATPS to surrounding soil after heating using a constant electrical current. Compared with previous buried sensors, the pluggable ATPS realizes recycle reutilizations of sensors and saves cost.
(2) With the advantages of high strength and large stiffness, ATPS can be used as a probe for the fast monitoring of highway slope and subgrade moisture. The detection depth and spatial resolution of the ATPS are flexible, which can be regulated based on application requirements.
(3) The results of the validation test indicated that the ATPS enables the detection of real-time changing soil moisture in the highway slope and subgrade after rainfall. For a naked subgrade slope, the most dangerous zones often occur at the connection between different construction materials. At these connections, cracks form easily and there is a significant increase in the soil hydraulic conductivity. Grassy cover (lawn) significantly prevents water infiltration during the first few rainfall minutes.
(4) The influence of the lawn on water infiltration depends on soil structure, hydraulic conductivity, and rainfall time. In some dangerous sites where the stability of highway slopes is susceptive to the soil moisture content, the ATPS should be installed in the hazardous zones to ensure that field monitoring data is collected in time.
